# Supplementary material for: Analysis of the Genome of the Heavy Metal Resistant and Hydrocarbon-Degrading Rhizospheric Pseudomonas qingdaonensis ZCR6 Strain and Assessment of Its Plant-Growth-Promoting Traits
Source: Int J Mol Sci. 2021 Dec 25;23(1):214. doi: 10.3390/ijms23010214 (PMC8745256; doi:10.3390/ijms23010214)
Supplement: Supplementary file 1 [file ijms-23-00214-s001.zip › ijms-1499682-supplementary.pdf]

Table S1. Accession numbers of the sequences of different type *Pseudomonas* spp. strains used in the MLSA phylogenetic analysis.

Strain	Genes		
	**16S rRNA**	***gyrB***	***rpoD***
P. aeruginosa	X06684	AB039386	AB039607
P. alkylphenolia	CP009048	HE577792	HE577794
P. brassicae	LC514379	LC514386	LC514382
P. graminis	Y11150	FN554187	FN554469
P. huaxiensis	MH428812	QKVL01000007	QKVL01000021
P. japonica	BBIR01000146	FZOL01000021	FZOL01000014
P. laurentiana	KY471137	BMQU01000001	BMQU01000017
P. plecoglossicida	AB009457	FN554218	FN554503
P. putida	AP013070	AB039451	AB039581
P. qingdaonensis	MG589917	MH758785	MH758786
P. rhizosphaerae	CP009533	FN554224	FN554510
P. taiwanensis	EU103629	FJ418634	HE577796

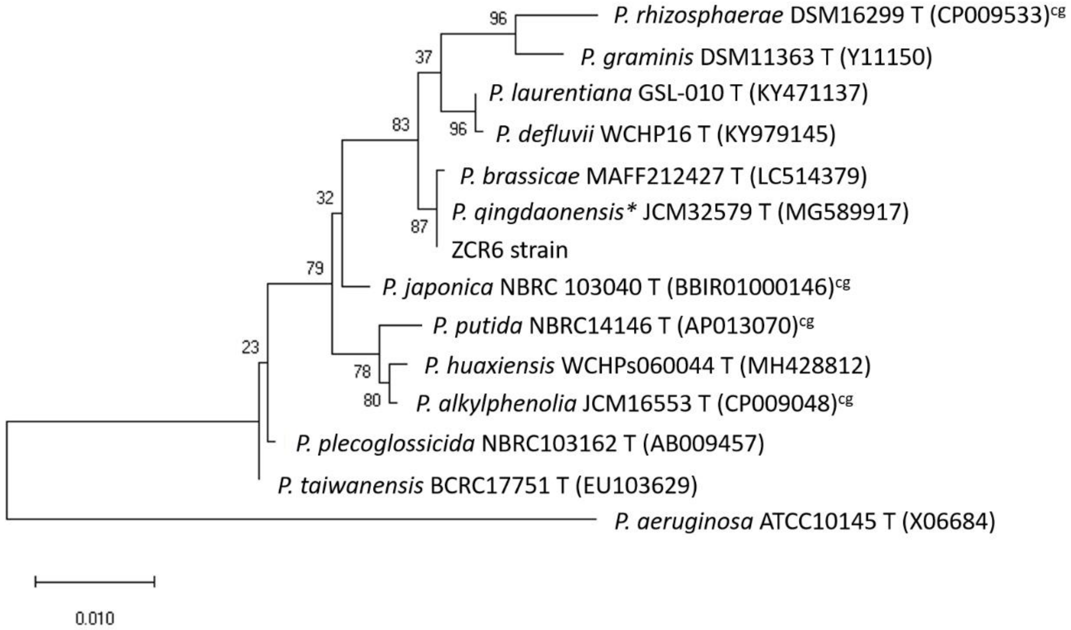

Figure S1. Phylogeny of type strains closely related to ZCR6 strain based on 16S rRNA gene sequence (accession numbers of sequences used in this analysis are given in brackets). All positions containing gaps or missing data were eliminated, which resulted in a 1387 bp sequence in the final dataset. Bootstrap values are represented at the branching points. The bar represents 0.010 substitutions per site. ^{cg} – sequence was extracted from complete genome sequence using Geneious Prime software; * strain JJ3^T with the highest ANI value.

Table S2. Secondary metabolites region recognized in the genome of the ZCR6 strain.

Region	Type	From	To	Most similar known cluster	Similarity
Region 1.1	Redox-cofactor	56.874	79.033	Lankacidin C	NRP+Polyketide 13%
Region 2.1	Thiopeptide, Lap	219.667	269.077		
Region 5.1	TransAT-PKS	77.087	134.533	Toblerol A	Polyketide 45%
Region 4.1	Betalactone	28.838	58.571		
Region 9.1	NAGGN	71.163	85.906		
Region 12.1	NRPS	1	65.143	Pyoverdin	NRP 17%
				Viscosin	NRP 45%
				Gacamide A	NRP: Lipopeptide 57%
				Orfamide B	NRP + Cyclic depsipeptide 50%
				Putisolvin	NRP 50%
				Anikasin	NRP 44%
Region 13.1	NRPS	1	29.5	Pyoverdin	NRP 11%
Region 30.1	NRPS	1	6.827	Rhizomide B	NRP 100%
Region 34.1	NRPS	1	3.686		

NRP - Nonribosomal peptides, NRPS - Nonribosomal peptide synthetases, NAGGN - N-acetylglutaminylglutamine amide

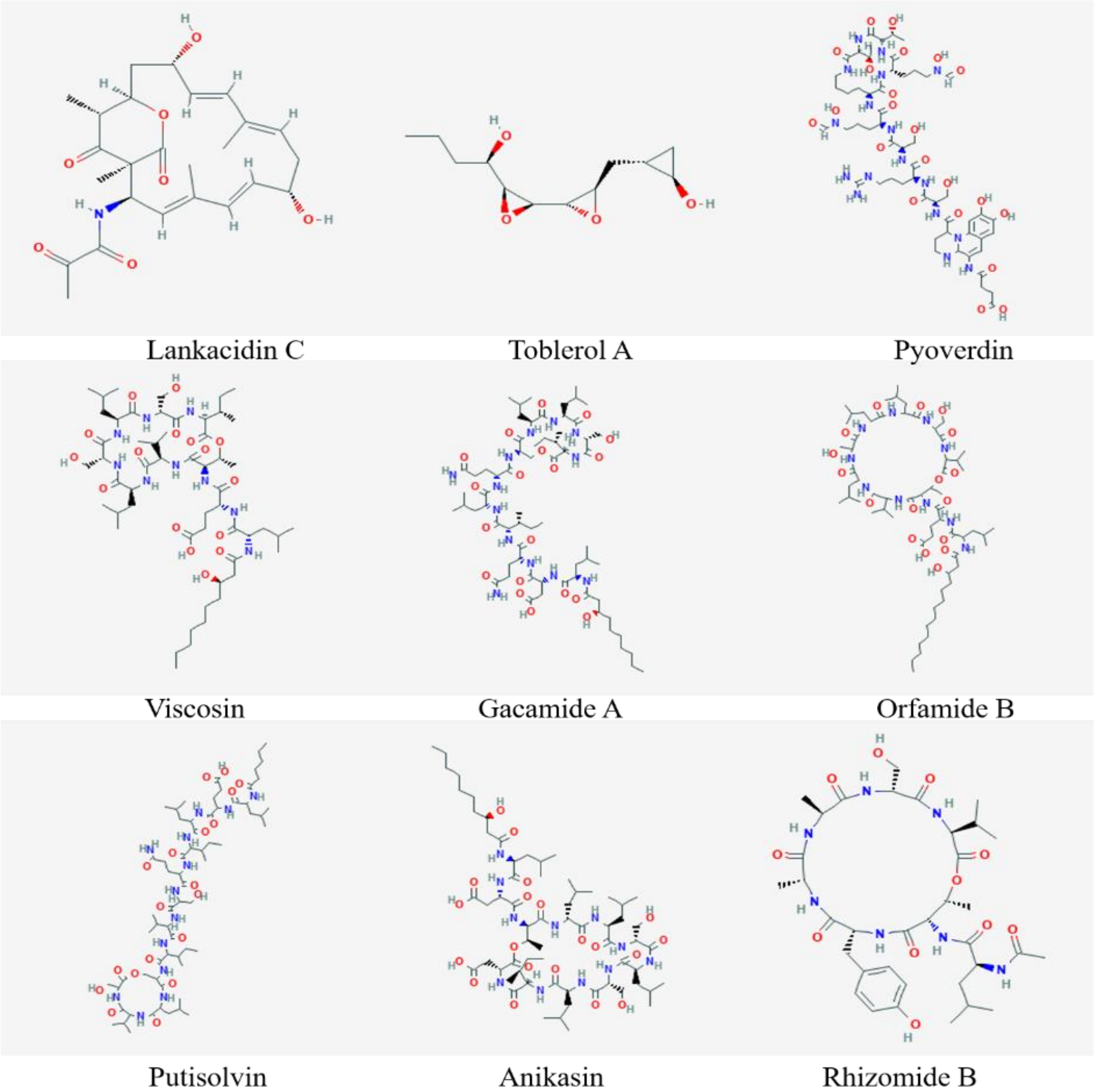

Figure S2. Predicted structures of secondary metabolites recognized for the ZCR6 strain. The structure was obtained from PubChem Compound (https://pubchem.ncbi.nlm.nih.gov/).
